# Parental Perception About Metered-Dose Inhalers and Nebulizers Differences Among Saudi Arabia

**DOI:** 10.7759/cureus.13548

**Published:** 2021-02-25

**Authors:** Abdullah A Alzayed, Amani S Alotaibi, Rahaf F Alfadhli, Renad A Alageel, Wejdan S Al-Saqat, Hussain A Alghadeer

**Affiliations:** 1 Pediatric, College of Medicine, Imam Mohammad Ibn Saud Islamic University, Riyadh, SAU; 2 Pediatric, College of Medicine, King Faisal University, Al-Ahsa, SAU

**Keywords:** asthma, pediatric, mdi, nebulizer, perception

## Abstract

Background

Asthma is a common public health issue in the pediatric population. The prevalence of asthma in children in Saudi Arabia is increasing. All asthmatic children with continuous symptoms should use controller medications. These medications if used correctly by the patients will diminish the symptoms and avoid exacerbations that lead to hospitalization. Perception of parents toward a particular device can affect the adherence rate.

Aim

Assessing the parental perception about metered-dose inhalers (MDIs) and nebulizers differences among the Saudi population.

Methods

A cross-sectional study was conducted to assess Parental perception about MDIs and nebulizers among Saudi parents with an asthmatic child. The data were collected from the parents and caregivers by using an online questionnaire and informed consent was obtained. The questionnaire was focused on demographic characteristics, knowledge, perception and practice of treating the asthmatic child. Data were analyzed through the Statistical Package for the Social Sciences (SPSS) version 25 (IBM Corp., Armonk, NY) and the results were considered statistically significant if P < 0.05.

Results

A total of 1,021 participants included in this study. This study found asthma is predominant in males (64.4%) and the most affected age is between 8 and 14 years (56.3%). More than half of the parents (58.7%) had a bachelor's degree and above. MDI is the most method used for controlling asthma (32.7%) and the majority of them (70.2%) thought there is a therapeutic difference between MDI and nebulizer. Providing instructions and information on usage MDI was received from 65.2%. Significant relationships were found between the level of satisfaction and receiving enough information about MDI and level of education.

Conclusion

This study found that asthma is more prevalent in males, where MDI is more common. Majority of the parents had thought that there is a difference in the therapeutic effects between MDI and nebulizer. They believed that nebulizer is more effective, less side effect and cheaper while MDI is easier to use.

## Introduction

Asthma is a chronic disorder and a common public health issue, especially among the pediatric population. Bronchial asthma is defined as a chronic medical condition that causes inflammation of the airways. The chronic inflammation will cause an increase in the hyper-responsiveness of the airways that results in breathlessness, chest tightness, recurrent episodes of wheezing and coughing especially in the early morning or at night. These episodes are generally connected with diffuse airflow obstruction that is usually reversible either developing without apparent external influence or with treatment [1]. The terms “chest allergy” and “dyspnea” are usually used instead of asthma in developing countries to avoid the social stigma related to the chronic nature of the disease [2].

The prevalence of asthma in children in Saudi Arabia is increasing rapidly [2] and fluctuates among various areas throughout the country. The highest prevalence among all reigns was in Alhofuf (33.7%) and the lowest prevalence was in Abha (9%) [3]. In Saudi Arabia, asthma has become one of the most common chronic disease, mostly related to the changes in lifestyle and modernization of Saudi society, the changes in eating habits, and excessive exposure to environmental factors such as dust, tobacco and sandstorms [2].

The National Heart, Lung and Blood Institute (NHLBI) of the National Institutes of Health has built up guidelines for the diagnosis and management of asthma (NHLBI, 1997) [4]. These guidelines state four things that should be done to control asthma: (a) regular assessment and monitoring of symptoms, (b) giving the appropriate medication, (c) taking control of environmental triggers and patient education, and (d) building relationship with families (NHLBI,1997) [4].

National guidelines state that all asthmatic children with continuous symptoms should use controller medications to achieve the greatest asthma control. These medications, if used correctly by the patients it will diminish the symptoms and avoid exacerbations that lead to hospitalization [5].

One study found that parents of asthmatic patients think the inhaler is easier to use when compared with the nebulizer, in the parent's opinion the nebulizer works better and a good system of delivery. The level of parent's education did not affect (P =0.584) the selection of the medication whether metered-dose inhalers (MDIs) or nebulizers. The reason for parental preference for nebulizer compared to MDIs were varied [6]. Moreover, it was found that misperceptions lead to ineffective behaviors and practices in the management of asthmatic child [2]. Also, cross-sectional study published in Aminu Kano Teaching Hospital, Kano in 2017 suggest by Structured questionnaires that parental education has a great impact in accepting the better kind of treating asthmatic child [7].

It is crucial to evaluate parental perception of MDIs and nebulizers because it has an impact on the adherence of the medication and it may be modified by building a better doctor-patient relationship and discussion. Therefore, our main objective of this study is to assess the parental perception about MDI and Nebulizers differences among the Saudi population.

## Materials and methods

Study design and population

Analytical cross-sectional survey was conducted during November to December 2020 to assess parental perception about MDIs and nebulizers The population of the study consisted of parents with asthmatic child in Saudi Arabia.

Sample size and sampling strategy

A sample size equal to 1,594 participants completed the survey.

Data collection

The data were collected through an electronic questionnaire distributed among parents with asthmatic child. Informed consent obtained from all the participants for completion of the survey considering the maximum privacy, safety and confidentiality. Inclusion criteria that all children diagnosed with asthma and using either MDI or nebulizer. Excluding parents for healthy children not using any kind of inhalers. The questionnaire included demographic characteristics, knowledge, perception and practice was assessed by descriptive statistics.

Data analysis

Data were analyzed through the Statistical Package for the Social Sciences (SPSS), version 25 (IBM Corp., Armonk, NY). Mean and standard deviation used to calculate the continuous data, frequencies, and percentages for categorical data and chi-square were considered statistically significant at P values <0.05.

Data management

Data is stored in a database on a safe computer within an encrypted file, and it is only shown to the research team. Privacy and confidentiality are maintained under all circumstances.

Ethical consideration and issues

For study protocol/study design/methodology: the study was approved by the medical ethics committee of Al-Imam Muhammad Ibn Saud Islamic University, Institution Review Board (IRB), RegistrationL HAPO-01-R-011 (ethical approval code: 98-2020).

## Results

In this study, 1,594 subjects completed the survey. Excluded participants were 35.9% that didn’t meet the criteria of this study. The study was completed by over 1021 participants.

Table 1 shows the demographical data of the participants. More than half of parents reported having asthmatic children between the age of 8 and 14 years (56.3 %) while 43.7 % were younger than seven years. The prevalence of asthma was predominant in males 64.4 % in ratio 2:1 (M:F). Majority of the participants 68.9% were between the age of 30 years or older while 31.1 % were between 19 and 29 years. Educational level of the most caregivers 58.7 % were bachelor degree or above education and 23.9% at level of high school. Lower percentage of them 17.4 % were at the level of primary or intermediate education.

**Table 1:** Demographic data of participants.

		N (%)
Age of child	<7	446 (43.7)
	8-14	575 (56.3)
Child's gender	Male	658 (64.4)
	Female	363 (35.6)
Age of caregiver	19-29 years	318 (31.1)
	30 years and above	703 (68.9)
Level of education	Primary/intermediate school level	178 (17.4)
	High school	244 (23.9)
	Bachelor degree and above	599 (58.7)

**Figure 1:**
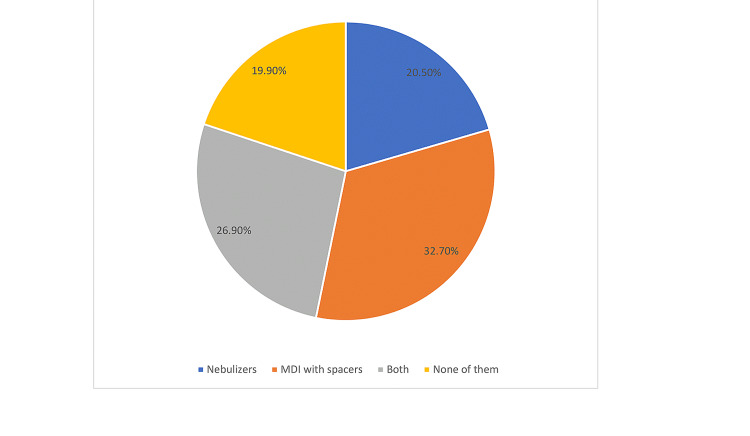
Treatment options which are providing to child as a treatment for bronchial asthma. MDI: metered-dose inhaler.

Figure 1 shows the treatment options for controlling asthma. About 32.7% of asthmatic children use MDI with spacers as treatment for their condition while 20.5% of them using of nebulizers. Around 26.9% indicated that using both modalities and 19.9% not using any device.

Table 2 shows the perception of parents of using MDI or nebulizer. Most of the participants 70.2% thought that there is a difference in the therapeutic effect between MDI and nebulizers in the treatment of asthma. More than half of them 65.2% indicated that they had received enough education from healthcare providers for adequate usage of MDI and 43.1% of them thought that usage of MDI is lifelong. 

**Table 2:** Perception of parents of using MDIs and nebulizers. MDIs: metered-dose inhalers.

	Yes, N (%)	No, N (%)
Do you think there is a difference in the therapeutic effect between MDIs and nebulizers in the treatment of bronchial asthma?	717 (70.2)	304 (29.8)
If you are using MDI, did you receive enough education from healthcare provider (nurse, doctor, health educator) on how to use it?	666 (65.2)	355 (34.8)
Do you believe that the asthmatic child who is using MDI will be lifelong dependent on this treatment?	440 (43.1)	581(56.9)

**Figure 2:**
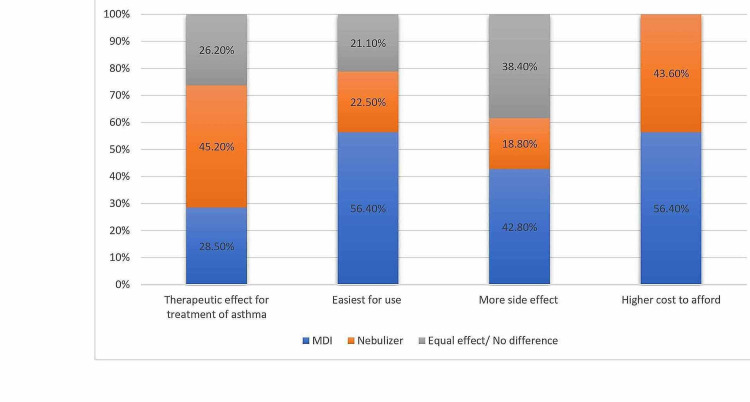
Difference of perception of parent between MDIs and nebulizers. MDIs: metered-dose inhalers.

Figure 2 shows the difference of perception of parents among MDI and nebulizer. The parents thought that nebulizers had a higher therapeutic effect for treatment asthma than MDI (45.2% vs 28.5%) while 26.2% did think there is a difference between them. On the other hand, participants indicated that MDI is easier to use (56.4% vs 22.5%). Moreover, around 42.80% thought that MDI has more side effects and 56.4% thought that MDI has higher cost.

Table 3 shows considering satisfaction and perception of parents about using MDIs and nebulizers with other factors like sociodemographic data. This study found that the mean score for MDI was 6.01 (SD = 2.59) which is lower than those with nebulizer where mean was 6.61 (SD = 2.63). A significant relationship was found between the age of child determines the level of satisfaction for both devices where satisfaction score increase in parents of older children (P = 0.000). However, the age of caregiver didn’t affect the satisfaction of parents about using both MDI or nebulizer (P = 0.621). Parents with male children would be significantly more satisfied with using MDI and nebulizer than those with female children (6.19 vs 5.69, 6.7 vs 6.43 P = 0.005). Regarding the educational level of parents is another determining factor for satisfaction especially in MDI. This represents that higher education level is associated with higher scores of satisfaction. Another significant relationship found between the education for the usage of MDI and level of satisfaction. Parents who received education for the usage of MDI are more satisfied (6.38 vs 5.28, P = 0.000). The perception of parents about safety, usage and effectiveness of two modalities have a significant effect on determining their satisfaction level. Thought of that the device is more effective, less side effect, easier to use and with low cost is associated with higher satisfaction scores. 

**Table 3:** Satisfaction of parents and their perception about the MDIs and nebulizers. *Significant at P-value <= 0.05. MDI: metered-dose inhaler.

		How satisfied are you with using the MDI, from 1 to 10?	How satisfied are you with using the nebulizer, from 1 to 10?	
		Mean (standard deviation)	Mean (standard deviation)	P-value
How old is your child	<7	5.61 (2.72)	6.30 (2.68)	0.000*
	8-14	6.31 (2.46)	6.84 (2.57)	
What is your child's gender?	Male	6.19 (2.57)	6.70 (2.57)	0.005*
	Female	5.69 (2.62)	6.43 (2.72)	
How old is the caregiver?	19-29 years	5.95 (2.72)	6.48 (2.72)	0.621
	30 years and above.	6.04 (2.54)	6.66 (2.58)	
What is your level of education?	Primary/intermediate school level	5.40 (2.81)	6.20 (2.82)	0.000*
	High school	6.00 (2.50)	6.69 (2.45)	
	Bachelor degree and above	6.21 (2.54)	6.70 (2.63)	
Did you receive enough education from healthcare providers (nurse, doctor, health educator) on how to use it?	Yes	6.38 (2.58)		0.000*
	No	5.28 (2.47)		
Which of the following do you think had more therapeutic effect for treatment of asthma?	MDI	6.41 (2.59)	6.38 (2.40)	0.001*
	Nebulizer	5.67 (2.48)	6.79 (2.72)	
	Equal effect	6.18 (2.74)	6.53 (2.68)	
In your opinion, which of the following is easier to use?	MDI	6.53 (2.38)	6.68 (2.45)	0.000*
	Nebulizer	5.04 (2.62)	6.53 (2.92)	
	No difference	5.66 (2.78)	6.50 (2.75)	
Which of the following modalities of treatment have more side effects?	MDI	5.71 (2.60)	6.60 (2.66)	0.001*
	Nebulizer	6.23 (2.54)	6.19 (2.78)	
	No difference	6.23 (2.60)	6.82 (2.49)	
Which of the following modalities has more cost to afford?	MDI	5.8 (2.60)	6.48 (2.66)	0.000*
	Nebulizer	6.28 (2.57)	6.77 (2.58)	

Table 4 shows the relationship between demographic factors of parents and children and the parental perception about MDI and nebulizer. In most variables, no significant correlation between these variables and demographic factors found except in the following variables. Age of children, age of care providers and education of parents have a significant effect on the perception of parents about ease of both nebulizer and MDI where parents of older children thought in higher extent that MDI is easier than nebulizer than those with younger children (P = 0.016). The same is found in other factors where higher educated parents thought that MDI is easier than nebulizer (P = 0.008). A significant relationship was found that younger parents had a perception that both tools were easy with no difference (0.001). Moreover, older and high educational levels had more beliefs that MDI didn’t relate with lifelong use in children. Gender of children has only effect on the perception of parents about the cost of modalities where parents of male children thought that the two modalities are costly than those with female children (67.2 % vs 32.8 % and 60.9 % vs 39.1 %, P = 0.037). 

**Table 4:** The relationship between demographic factors and perception of parents. *Significant at P-value <= 0.05. MDI: metered-dose inhaler.

		How old is your child		What is your child's gender?		How old is the caregiver?		What is your level of education?		
		<7	8-14	Male	Female	19-29 years	30 years and above	Primary	High school	Bachelor degree
Do you think there is a difference in the therapeutic effect between MDI and nebulizers in the treatment of bronchial asthma?	Yes	42.3%	57.7%	65.0%	35.0%	30.3%	69.7%	16.2%	23.2%	60.7%
	No	47.0%	53.0%	63.2%	36.8%	33.2%	66.8%	20.4%	25.7%	53.9%
	P-value	0.159		0.575		0.351		0.122		
Which of the following do you think had more therapeutic effect for treatment of asthma?	MDI	46.4%	53.6%	62.5%	37.5%	30.9%	69.1%	17.5%	20.3%	62.2%
	Nebulizer	43.9%	56.1%	66.0%	34.0%	28.4%	71.6%	18.4%	25.8%	55.8%
	Equal effect	40.3%	59.7%	63.8%	36.2%	36.2%	63.8%	15.7%	24.6%	59.7%
	P-value	0.345		0.605		0.088		0.361		
If you are using MDI, did you receive enough education from healthcare provider (nurse, doctor, health educator) on how to use it?	Yes	41.6%	58.4%	65.5%	34.5%	32.4%	67.6%	17.0%	24.0%	59.0%
	No	47.6%	52.4%	62.5%	37.5%	28.7%	71.3%	18.3%	23.7%	58.0%
	P-value	0.065		0.352		0.224		0.856		
In your opinion, which of the following is easier to use?	MDI	39.9%	60.1%	66.0%	34.0%	28.3%	71.7%	15.5%	21.5%	63.0%
	Nebulizer	50.4%	49.6%	63.9%	36.1%	28.3%	71.7%	18.3%	30.4%	51.3%
	No difference	46.5%	53.5%	60.9%	39.1%	41.9%	58.1%	21.9%	23.3%	54.9%
	P-value	0.016*		0.412		0.001*		0.008*		
Do you believe that the asthmatic child who is using MDI will be lifelong dependent on this treatment?	Yes	46.8%	53.2%	65.2%	34.8%	35.2%	64.8%	20.9%	23.9%	55.2%
	No	41.3%	58.7%	63.9%	36.1%	28.1%	71.9%	14.8%	23.9%	61.3%
	P-value	0.079		0.650		0.014*		0.031*		
Which of the following modalities of treatment have more side effects?	MDI	47.4%	52.6%	63.6%	36.4%	29.1%	70.9%	16.9%	21.1%	62.0%
	Nebulizer	41.7%	58.3%	67.7%	32.3%	33.3%	66.7%	21.4%	23.4%	55.2%
	No difference	40.6%	59.4%	63.8%	36.2%	32.4%	67.6%	16.1%	27.3%	56.6%
	P-value	0.117		0.577		0.449		0.132		
Which of the following modalities has more cost to afford?	MDI	45.7%	54.3%	67.2%	32.8%	29.0%	71.0%	16.8%	23.1%	60.1%
	Nebulizer	41.1%	58.9%	60.9%	39.1%	33.9%	66.1%	18.2%	24.9%	56.9%
	P-value	0.147		0.037*		0.091		0.586		

## Discussion

Asthma is a worldwide disease and considered one of the most common diseases in pediatric populations [1]. The prevalence is increasing rapidly in Saudi Arabia [2]. In enhancing the quality of life of asthmatic children, asthma care has been accomplished, such as offering appropriate medication and preventive steps. Asthma treatment according to guidelines fails frequently [4]. Low adherence to MDI treatment in children with asthma is one of the main reasons why asthma is still associated with significant morbidity, and goals set in the GINA guidelines are frequently not met [1,8]. Several factors lead to poor adherence, one of them the knowledge and perception of parents toward this condition affect the treatment strategies in children and more important the adherence to prescribed treatments [9]. Therefore, it is very important for regular assessment of this perception and develops methods for improving them. The aim of this study is to assess the parental perception about MDI and Nebulizers differences among the Saudi population.

One of the important findings in this study is that the prevalence of asthma was higher among male than female children with the ratio of about 2:1. The previous studies showed that the prevalence of asthma is higher in female than male as the study of Horaib 2018, who had found that asthma prevalence among girls (14.4%) was higher than boys (12.4%) [10] and the study of Aliyu 2018, where the gender of the asthma patients were 55 (46.2%) males and 64 (53.8%) females with male to female ratio of 1:1.2 [7]. The high prevalence of male asthmatic children can explain why parents of male children complain from cost of the two modalities than parents of female children. Most of the patients 32.7% were using MDI with spacers as treatment for their condition while 20.5 % using nebulizer and 26.9 % indicated the use of both modalities. This is different from the reported study of Aliyu where 72.3% of the respondents would accept MDI if prescribed [7].

The parents thought that there is a difference between MDIs and nebulizers with some preference of one tool over the other in some aspects like nebulizer is more effective, less side effect and cheaper than MDI. On the other hand, they stated that MDI is easier to be used. Around 43.1% of the parents had myths that since started MDI, would lead to use it for lifelong which is higher than reported in other studies as Aliyu who had found that 51.5% of parents believed the use of MDI will make asthma protracted [11]. The fining of this study was similar to the results of Bosley et al. who reported their respondents stating that asthma medications were addictive and may be ineffective on prolonged use [11]. These myths were related to some demographic factors as parents of older children and those with higher education thought that MDI is easier to while older and more educated parents had more beliefs that MDI do not relate with lifelong use in children.

A significant relationship was found between the parent perception about the medication and their satisfaction with it and this will affect the adherence of children to medication where thought of that the device is more effective, less side effect, easier to use and with low cost is associated with higher satisfaction scores. Moreover, other factors included a higher level of education would reflect a high score of satisfaction which is similar to Aliyu [7]. Providing instruction and education of parents about how to use MDI, will increase the level of satisfaction and adherence. This is an indication of the importance of providing the parents of asthmatic patients with information about different treatment apparatus and proper information about the disease that will help in increase the rate of adherence providing more control on asthma of their children.

Limitations of this study are people may not answer the questions honestly and precisely, this may affect the data analysis and may result in bias. Also, understanding the questions is very important to limit and minimize errors. Furthermore, uneducated population could also result in bias due to limited knowledge about the questions and the title of the study.

## Conclusions

This study concluded that asthma is more prevalent in females where MDI was the most common treatment used by children. Moreover, most parents had thought that there is a difference between MDI and nebulizer where they thought that nebulizer is more effective, less side effect and cheaper than MDI while they thought that MDIs are easier to be used than nebulizers. Some inadequate perception including that MDI may lead to remain on medication affect the satisfaction of parents. More investigation should be done to study the effect of satisfaction level of parent about one instrument of adherence rate of their children.
